# Deep Brain Stimulation Modulates Multiple Abnormal Resting-State Network Connectivity in Patients With Parkinson’s Disease

**DOI:** 10.3389/fnagi.2022.794987

**Published:** 2022-03-21

**Authors:** Yutong Bai, Yu Diao, Lu Gan, Zhizheng Zhuo, Zixiao Yin, Tianqi Hu, Dan Cheng, Hutao Xie, Delong Wu, Houyou Fan, Quan Zhang, Yunyun Duan, Fangang Meng, Yaou Liu, Yin Jiang, Jianguo Zhang

**Affiliations:** ^1^Department of Neurosurgery, Beijing Tiantan Hospital, Capital Medical University, Beijing, China; ^2^Department of Radiology, Beijing Tiantan Hospital, Capital Medical University, Beijing, China; ^3^Department of Neurosurgery, Beijing Neurosurgical Institute, Beijing, China; ^4^Beijing Key Laboratory of Neurostimulation, Beijing, China

**Keywords:** Parkinson’s disease, deep brain stimulation, resting-state network, functional connectivity, power spectra, frontoparietal network

## Abstract

**Background:**

Deep brain stimulation (DBS) improves motor and non-motor symptoms in patients with Parkinson’s disease (PD). Researchers mainly investigated the motor networks to reveal DBS mechanisms, with few studies extending to other networks. This study aimed to investigate multi-network modulation patterns using DBS in patients with PD.

**Methods:**

Twenty-four patients with PD underwent 1.5 T functional MRI (fMRI) scans in both DBS-on and DBS-off states, with twenty-seven age-matched healthy controls (HCs). Default mode, sensorimotor, salience, and left and right frontoparietal networks were identified by using the independent component analysis. Power spectra and functional connectivity of these networks were calculated. In addition, multiregional connectivity was established from 15 selected regions extracted from the abovementioned networks. Comparisons were made among groups. Finally, correlation analyses were performed between the connectivity changes and symptom improvements.

**Results:**

Compared with HCs, PD-off showed abnormal power spectra and functional connectivity both within and among these networks. Some of the abovementioned abnormalities could be corrected by DBS, including increasing the power spectra in the sensorimotor network and modulating the parts of the ipsilateral functional connectivity in different regions centered in the frontoparietal network. Moreover, the DBS-induced functional connectivity changes were correlated with motor and depression improvements in patients with PD.

**Conclusion:**

DBS modulated the abnormalities in multi-networks. The functional connectivity alterations were associated with motor and psychiatric improvements in PD. This study lays the foundation for large-scale brain network research on multi-network DBS modulation.

## Introduction

Deep brain stimulation (DBS) is a promising treatment in patients with moderate-to-advanced Parkinson’s disease (PD) (Krauss et al., 2021; Yin et al., 2021b). DBS can significantly improve motor and non-motor symptoms (Mansouri et al., 2018; Cartmill et al., 2021; Diao et al., 2021; Yin et al., 2021a), although the neuromodulation mechanism of DBS is still unclear.

Studies of DBS-modulated abnormal neurocircuits have mainly focused on the motor network. In neuroimaging studies, it is generally believed that functional connectivity declines within sensorimotor networks (SMNs) in patients with PD, when compared with healthy controls (HCs) (Suo et al., 2017; Ji et al., 2018). Functional connectivity changes in the SMN and basal ganglia network correlate with motor severity (Manza et al., 2016). DBS increased the effective connectivity in the direct pathways, which led to decoupling of the functional connectivity in the subthalamic nucleus (STN) (Kahan et al., 2019). In electrophysiological studies, similar results have also been reported, namely, DBS excited local neuron activity, which inhibited synchronization between basal ganglia and the primary motor cortex (M1) (Wichmann and DeLong, 2016). All these studies showed that DBS improved the efficiency of information transmission in the thalamus (Molnar et al., 2005). This phenomenon increases the excitability of the primary motor cortex (M1), to improve motor symptoms (Johnson et al., 2020).

Still, studies of modulating abnormal neurocircuits in multi-networks by DBS are limited. It has been reported that patients with PD had multi-network impairments when compared with controls (Mohan et al., 2016). The non-motor symptoms in PD are closely related to functional connectivity changes in multi-networks (Tinaz, 2021). Clinical studies showed that DBS significantly improved PD non-motor symptoms (Petry-Schmelzer et al., 2019; Diao et al., 2021; Yin et al., 2021a). However, the modulation patterns of DBS in multi-networks remain unclear. The responses between dopaminergic drugs and DBS in symptom improvements are comparable (Mueller et al., 2018). Previous studies showed that dopaminergic drugs (medication on) partially normalized PD-related functional connectivity patterns in multi-networks when compared with nondrug states (medication off) (Tinaz, 2021). Accordingly, we hypothesized that DBS may also modulate functional connectivity in a multi-network manner.

In this study, we aimed to characterize the multiple resting-state network (RSN) alteration patterns using DBS modulation. We obtained resting-state functional MRI (rs-fMRI) data from patients with PD after stable DBS, by comparing differences within and between networks among different groups.

## Materials and Methods

### Participants

A total of 40 patients from the Department of Neurosurgery, Beijing Tiantan Hospital, were enrolled in this study. The inclusion criteria were as follows: (1) diagnosed with idiopathic PD according to the UK Brain Bank Clinical Criteria, (2) implanted with bilateral STN-DBS (Medtronic 3389; Medtronic, Dublin, Ireland) for at least 3 months, (3) Hoehn-Yahr (H&Y) stage 2.5–4.0 in medication-off, and (4) preoperative medication improvement rate higher than 30%. The exclusion criteria were as follows: (1) severe head tremors, (2) combining with other neurological diseases, (3) unilateral lead implantation, and (4) implantation of an internal pulse generator (IPG) in the right chest. We also enrolled 28 age-matched HCs. This study was approved by the Institutional Review Board of Beijing Tiantan Hospital of Capital Medical University (Approval Number: KY 2018-008-01). All participants provided written informed consent. More than 20 subjects for PD and HC were enrolled because a sample size ≥ 20 was recommended for sufficient reliability in fMRI studies (Thirion et al., 2007). The experimental protocol adhered to the tenets of the Declaration of Helsinki.

### Clinical Evaluation

The flowchart of this study is illustrated in Supplementary Figure 1. The optimal parameters were programmed by experienced clinicians within 1 week of scans. Patients were required to withdraw their medications overnight on the day before scanning. On the day of scanning, patients were assigned by using a pseudorandom method into two balanced scanning pathways. In one pathway, patients first received scanning in the DBS-on state, while the motor functions were evaluated immediately after scanning (stim-on/med-off). Then, the DBS was switched off, and the patients waited for 2 h or until the motor symptoms reappeared. Patients received scans for the second time in the DBS-off state, while the motor functions were evaluated immediately after scanning (stim-off/med-off). In the other pathway, the order of scanning was swapped, to minimize bias from poststimulus effects (Zhang et al., 2021).

Motor functions were evaluated using the Movement Disorder Society Unified Parkinson’s Disease Rating Scale part III (MDS-UPDRS-III). MDS-UPDRS-III contained four domains (Stenmark Persson et al., 2021), namely, tremor (items 15–18), rigidity (item 3), bradykinesia (items 2, 4–8, and 14), and axial (items 1 and 9–13). The Hamilton Depression Scale (HAM-D) and the Hamilton Anxiety Scale (HAM-A) were used to assess the severity of both preoperative and postoperative mood disorders.

### MRI Acquisition

Participants were scanned using a 1.5-T GE SIGNA Explorer MRI scanner (General Electric, San Ramon, CA, United States). We acquired the rs-fMRI data in two states as described above. Structural images were conducted by using the magnetization-prepared rapid acquisition gradient echo sequence: repetition time (TR) = 1,146 ms, echo time (TE) = 4.97 ms, flip angle (FA) = 12°, voxel size = 1 mm × 1 mm × 0.7 mm, slices = 286, field of view (FOV) = 240 mm × 240 mm, and matrix size = 256 mm × 256 mm. The blood-oxygen-level-dependent (BOLD) image was obtained using the following echo-planar imaging sequence: TR = 3,000 ms, TE = 40 ms, FA = 90°, acquisition matrix = 64 × 64, number of slices = 36, voxel size = 3.75 mm × 3.75 mm × 4 mm, slice gap = 1 mm, FOV = 240 mm × 240 mm, and scanning time ≈ 7 min.

### Artifact Filling and Preprocessing of fMRI Data

**FIGURE 1:**
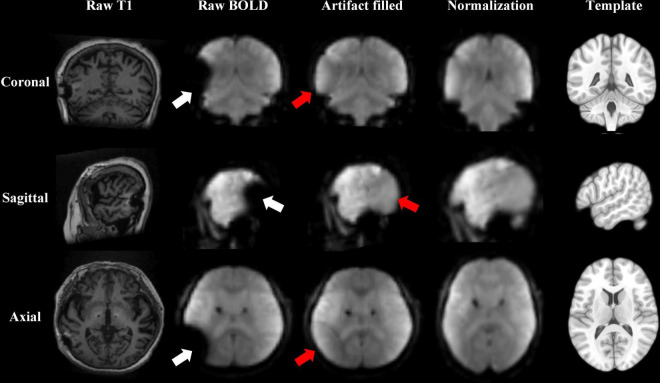
Resting-state fMRI data preprocessed after the deep brain stimulation (DBS) artifact was filled. An enantiomorphic normalization method was used to fill the artifacts of the raw blood-oxygen-level-dependent (BOLD) data, to improve the accuracy of normalization to the Montreal Neurological Institute (MNI) space. The white arrow shows the artifact lesion. The red arrow shows the filled lesion. The results are displayed from coronal, sagittal, and axial views in three rows. The columns show the raw T1 image, raw BOLD image, and BOLD image after the artifacts were filled; the BOLD image after normalization; and the MNI template from left to right.

The MRI scans after DBS suffered from magnetic susceptibility artifacts caused by the DBS apparatus, which reduced the accuracy of normalization (Holiga et al., 2015). All patients’ IPG were in the left chest, so all artifacts were in the left brain. The artifact areas included the partial left inferior parietal lobe, left temporal lobe, left occipital lobe, and left cerebellum. We used enantiomorphic normalization methods (mirror image normalization) to fill the artifact. The signal from the contralateral region (symmetry around the midline), specifically, is filled to the artifact region (Nachev et al., 2008; Yourganov et al., 2016; Keller et al., 2017). Thus, the artifact-filled images were better normalized to the Montreal Neurological Institute (MNI) space (Figure 1). The filled signals in these artifact regions were excluded for further analysis.

The artifact-filled images were preprocessed using SPM12^1^. Functional images were preprocessed according to a standard pipeline: (a) the first five time points (15 s) were removed; (b) scans were slice-time-corrected to the median (35th) slice in each TR; and (c) scans were then realigned to create a mean realigned image. Participants with head motions exceeding 3 mm or 3° of rotation in any direction were excluded. (d) Two-step normalization: T1 structural images were co-registered to rs-fMRI images using a nonlinear image registration approach and to automatically segment the brain into different ingredients. (e) Scans were spatially smoothed using 6 mm × 6 mm × 6 mm full width at half maximum (FWHM).

### Independent Component Analysis

Single-subject and group-level independent component analysis (ICA) was conducted using the Infomax algorithm using Group ICA of the fMRI Toolbox (GIFT V4.0)^2^. The number of independent components (ICs) were estimated using the minimum description length (MDL) criteria, and 20 ICs were extracted for each subject (Calhoun et al., 2001). Stability was assessed using ICASSO with 20 repetitions (Himberg et al., 2004). After back reconstruction, the spatial components were converted to *z*-scores in each single subject (*z*-maps). The components were identified by spatially sorting all components with the healthy volunteer anatomy masks of RSNs (Smith et al., 2009). Default mode network (DMN), SMN, right frontoparietal networks (r-FPN), salience network (SN), and left frontoparietal network (l-FPN) were extracted for further analyses (Supplementary Figure 2). The *z*-maps of selected components were submitted to a group-specific one-sample *t*-test, with cluster-wise false discovery rate (FDR) correction for multiple comparison, to describe each selected RSNs. For each network, the utility of spectral group comparison in the GIFT was used to compare the difference of power spectra of 0.01–0.08 Hz between groups. The functional connectivity among networks was also compared by using the MANCOVAN toolbox in Group ICA.

### Region of Interest Identification and Analysis of Functional Connectivity Among Resting-State Networks

To further observe the region-to-region connectivity, region of interest (ROI)-based connectivity analyses were performed. After temporally detrend, band-pass filter (0.01–0.08 Hz) and regress out covariates (white matter, cerebrospinal fluid (CSF) signal, and head motion), fifteen ROIs in the five abovementioned networks were selected (Yeo et al., 2014), including the medial prefrontal cortex (mPFC), posterior cingulate cortex (PCC), right inferior parietal lobule (r-IPL) in DMN, supplementary motor area (SMA), left primary motor cortex (l-M1), and right primary motor cortex (r-M1) in the SMN; anterior cingulate cortex (ACC), left insula (l-INS), and right insula (r-INS) in the SN; left premotor area (l-PMA), left dorsolateral prefrontal cortex (l-dlPFC), and left posterior parietal cortex (l-PPC) in the l-FPN; right premotor area (r-PMA), right dorsolateral prefrontal cortex (r-dlPFC), and right posterior parietal cortex (r-PPC) in the r-FPN.

For each ROI, BOLD signal time courses in each scan were extracted within a 6-mm sphere with the center localized at the peak voxel (the highest *t*-value after one-sample *t*-test for its component) (Supplementary Figure 3). Correlations among ROIs were calculated using Pearson’s correlation. For each subject in each scan, there was a matrix containing all the correlation coefficients (*r*-values) among the ROIs. The *r*-value matrices among HC, PD-off, and PD-on were compared, with age and sex regressed as covariates.

### Resting-State Network Behavioral Correlation

The correlation between functional connectivity changes and the clinical scores’ change rate (MDS-UPDRS-III total score; tremor, rigidity, bradykinesia, and axial subscores; HAM-A and HAM-D scores) was assessed using Pearson’s correlation. A univariable linear regression model was used to draw the best-fit line with the threshold of *P* < 0.05.

The motor change rate was calculated as follows:

$$\frac{\left(\mathrm{PD}-\mathrm{off}\,\mathrm{scores}\right)-\left(\mathrm{PD}-\mathrm{on}\,\mathrm{scores}\right)}{\left(\mathrm{PD}-\mathrm{off}\,\mathrm{scores}\right)}\times 100\%$$

The mood change rate was calculated as follows:

$$\frac{\left(\mathrm{preoperative}\,\mathrm{scores}\right)-\left(\mathrm{postoperative}\,\mathrm{scores}\right)}{\left(\mathrm{preoperative}\,\mathrm{scores}\right)}\times 100\%$$

### Statistical Analysis

Data were presented as mean ± SD or median (Q1, Q3) for continuous variables and percentage for binary variables. All variables were tested for normality by using the Anderson-Darling normality test. Comparisons between groups (HC vs. PD-off and HC vs. PD-on) were performed by the independent *t*-tests for continuous variables with the normal distribution and by the Mann-Whitney *U* test for continuous variables with the skew distribution. Comparisons of repeated measurements (PD-off vs. PD-on) were performed by the paired *t*-tests for continuous variables with the normal distribution and by the Wilcoxon matched-pairs signed-rank test for continuous variables with the skew distribution. The chi-square test was used for binary variables, and Pearson’s correlation was used for correlation analysis. The multiple comparison test was performed by FDR, and the corrected *P* < 0.05 was considered significant. All statistical analysis was conducted using SPSS 24 (IBM, Chicago, IL, United States) and Python 3. Images were drawn using GraphPad Prism 9.0 (GraphPad Software, San Diego, CA, United States).

## Results

### Clinical Motor and Psychology Findings

All participants were right-handed, without resting head tremors. A total of 17 patients were excluded, including 13 with excessive head movements (one in the HC group), and 4 intolerant to scanning. The remaining 24 patients with PD (62.5 ± 7.9 years, 15 men) and 27 HCs (61.6 ± 4.6 years, 14 men) were included for further analyses. Individual data for all patients with PD are listed in Supplementary Table 1. There was no significant difference in age and sex between the HCs and patients with PD. In patients with PD, the disease duration was 10.9 ± 3.2 years, the H&Y stage was 2.9 ± 0.2 at med-off preoperatively, and the levodopa equivalent doses were 661.6 ± 258.7 mg. The mean follow-up was 18.0 ± 17.6 months after DBS surgery. The comparison between baseline characteristics is shown in Table 1. DBS significantly improved not only overall motor performance and all motor subscores but also depression and anxiety.

**TABLE 1:** Baseline characteristics of the enrolled sample.

	*Patients (n = 24)*	*Controls (n = 27)*	*P-value*
*Age*	*62.5 ± 7.9*	*61.6 ± 4.6*	*0.617*
*Gender / Man*	*62.5%*	*51.9%*	*0.443*
*Disease Duration*	*10.9 ± 3.2*	*-*	
	**PD-off^a^**	**PD-on^a^**	

MDS-UPDRS-III	44.7 ± 16.3	22.3 ± 9.9	< 0.001*
Tremor	8.4 ± 5.8	3.5 ± 3.0	< 0.001*
Rigidity	7.5 ± 2.7	3.4 ± 2.0	< 0.001*
Bradykinesia	20.5 ± 8.9	10.4 ± 5.7	< 0.001*
Axial	9.3 ± 4.8	5.4 ± 3.4	< 0.001*
HAM-D^b^	16.9 ± 9.4^c^	12.8 ± 7.9^d^	< 0.001*
HAM-A^b^	16.9 ± 10.1^c^	11.8 ± 6.4^d^	< 0.001*

*Data were presented as the mean ± SD. PD-on and PD-off indicate the DBS status.*

*Statistics significant difference.

*^a^All data were collected during medication withdrawn for at least 12 h.*

*^b^HAM-D and HAM-A were collected in 20 patients.*

*^c^Preoperative scores.*

*^d^Last follow-up scores.*

### Identification and Power Spectra Changes Among Resting-State Networks

**FIGURE 2:**
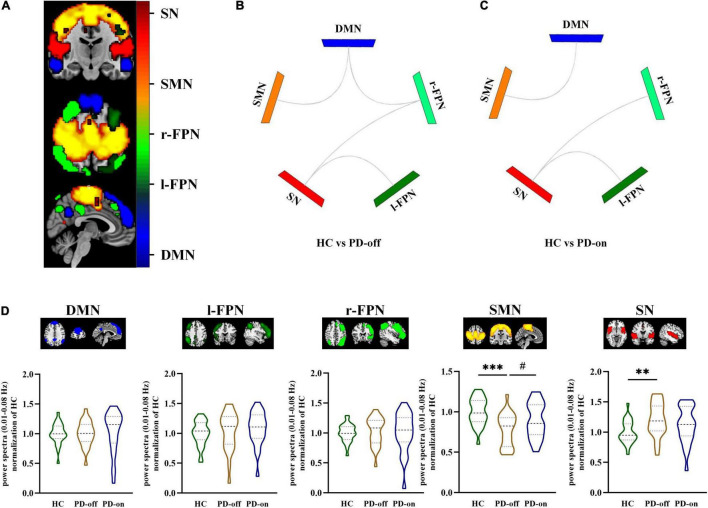
Selected resting-state networks (RSNs) and comparisons between groups in network level. **(A)** Selected RSNs (e.g., HC group): DMN, l-FPN, r-FPN, SMN, and SN (distinguished by colors, a one-sample *t*-test was used, and the threshold of the display was set to the cluster-level false discovery rate (FDR)-corrected *P* < 0.05). Different functional connectivities of selected networks were compared between **(B)** HC and PD-off and **(C)** HC and PD-on (significant differences were shown in gray line); no significant differences were seen between PD-off and PD-on. **(D)** Power spectra (0.01–0.08 Hz) differences between HC, PD-off, and PD-on within each network. Data are represented as the median (Q1, Q3). ^*,#^*P* < 0.05; ^**^P < 0.01; ^***^P < 0.001 (*compared with controls, ^#^compared with PD-off). HC, healthy control; PD, Parkinson’s disease; SMN, sensorimotor networks; DMN, default mode network; FPN, frontoparietal network; SN, salience network.

Five components were identified as RSNs of interest. These RSNs were DMN, l-FPN, r-FPN, SMN, and SN (Figure 2A, *P* < 0.05, FDR-corrected). The composition and location of each RSN were similar to the templates. The components in each group are shown in Supplementary Figure 2. The power spectra in SMN significantly decreased in PD-off compared with HC, while PD-on was significantly increased (HC vs. PD-off: −0.22, *P* < 0.001; PD-on vs. PD-off: −0.12, *P* = 0.030). The power spectra in SN significantly increased in PD-off compared with HC, with a decreasing trend seen in the PD-on (HC vs. PD-off: 0.21, *P* = 0.009; PD-on vs. PD-off: −0.09 *P* = 0.339). No significant difference was found in other networks (Figure 2D).

### Functional Network Connectivity Changes Among Resting-State Networks

In network analysis, compared with HC, PD-off showed decreased connectivity in DMN/SMN (*t* = 3.81, *P* < 0.001), DMN/r-FPN (*t* = −3.16, *P* = 0.003), SN/r-FPN (*t* = 4.03, *P* < 0.001), and SN/l-FPN (*t* = 3.68, *P* < 0.001), while PD-on showed decreased connectivity in DMN/SMN (*t* = 2.63, *P* = 0.011), SN/r-FPN (*t* = 3.20, *P* = 0.002), and SN/l-FPN (*t* = 3.56, *P* = 0.001). No significant difference was found in network connectivity between PD-on and PD-off (Figures 2B,C).

**FIGURE 3:**
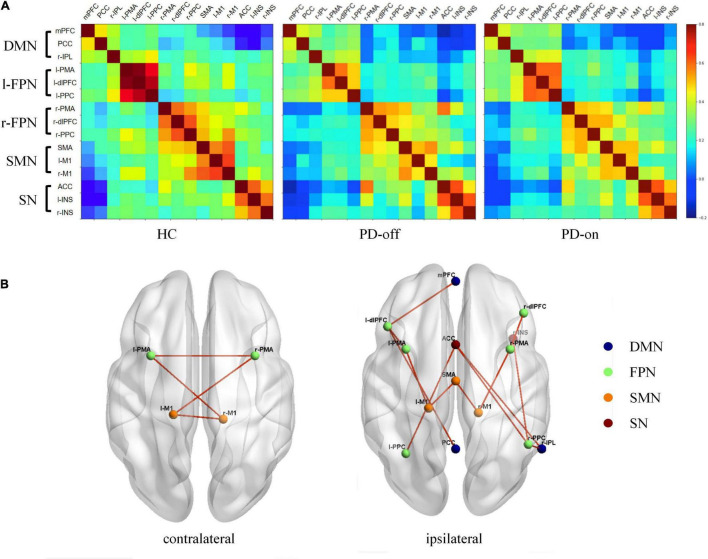
Different functional connectivities of selected regions of interest (ROIs) among HC, PD-off, and PD-on. **(A)** Functional connectivity matrix from selected ROIs for HC, PD-off, and PD-on. The color bar indicates the correlation coefficients between ROIs on the right. **(B)** Different functional connectivities of contralateral (left) and ipsilateral (right) among the three groups are shown separately in the three-dimensional view from the superior perspective. Spheres are shown as ROIs from networks with legends in the right. Lines denote significant differences (*P* < 0.05, FDR-corrected).

**FIGURE 4:**
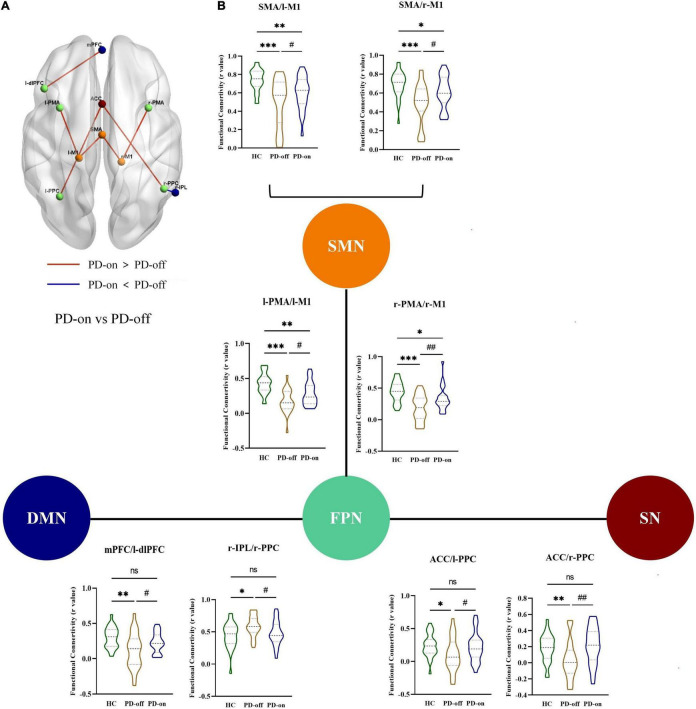
DBS reversed the abnormal functional connectivity in patients with PD. **(A)** Interstate analyses of abnormal functional connectivities. The connections shown in light red represent PD-on > PD-off; dark blue connections refer to PD-off > PD-on. **(B)** Additionally, these results were presented in the violin graph among the networks. The bars represent the median (Q1, Q3). ^*,#^*P* < 0.05; *^*,##^*P* < 0.01; ^***^P < 0.001 (*compared with controls, ^#^compared with PD-off).

Furthermore, in the ROI analysis, the mean functional connectivity matrix constructed by 15 ROIs for HC, PD-off, and PD-on was calculated by averaging the individual matrices and was shown in Figure 3A. A significantly changed functional connectivity between HC and PD-off is shown in Figure 3B (left, contralateral results; right, ipsilateral results). Most of the abnormal functional connectivities decreased in PD-off compared with HC, except the functional connectivity in r-PPC/r-IPL. The abnormal functional connectivity reversed by DBS is shown in Figure 4. All reversed functional connectivities were ipsilateral. The functional connectivity increased within SMN (SMA/l-M1 and SMA/r-M1), between FPN and SMN (l-PMA/l-M1 and r-PMA/r-M1), SN (l-PPC/ACC and r-PPC/ACC), and DMN (l-dlPFC/mPFC). The functional connectivity decreased between r-IPL/r-PPC. The statistical measures of the abovementioned functional connectivity are shown in Supplementary Table 2. Supplementary Figure 4 shows the abnormal functional connectivity, which could not be reversed by DBS. All results were FDR-corrected.

### Behavioral Correlations of Resting-State Networks

**FIGURE 5:**
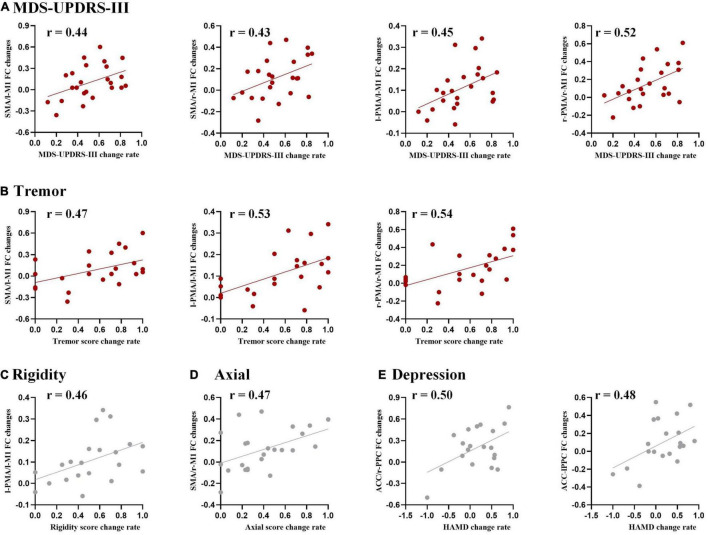
Correlation analysis between functional connectivity changes and DBS improvements for **(A)** Movement Disorder Society Unified Parkinson’s Disease Rating Scale part III (MDS-UPDRS-III), **(B)** tremor, **(C)** rigidity, **(D)** axial, and **(E)** the Hamilton Depression scale (HAM-D). The threshold was set to uncorrected *P* < 0.05. Results surviving to FDR correction were shown in red.

The functional connectivity changes between ROIs were correlated with the change rate of MDS-UPDRS-III total scores, subscores in each domain (tremor, rigidity, bradykinesia, and axial), and HAM-D and HAM-A scores (Figure 5).

Specifically, a positive correlation with the change rate of MDS-UPDRS-III total score was found in the functional connectivity changed in SMA/l-M1, SMA/r-M1, l-PMA/l-M1, and r-PMA/r-M1. Positive correlations with the change rate of tremor subscores were observed in the functional connectivity changes in r-PMA/r-M1, l-PMA/l-M1, and SMA/l-M1.

We also observed some tendency results, with uncorrected *P* < 0.05, but not survived after FDR correction, including (1) the change rate of rigidity subscore was a positive correlation with the functional connectivity changes in l-PMA/l-M1; (2) the change rate of axial subscore was a positive correlation with the functional connectivity changes in SMA/r-M1; and (3) the change rate of HAM-D was a positive correlation with l-PPC/ACC and r-PPC/ACC.

## Discussion

The first finding of this study was that SMN was a key network in DBS modulation, which involved modulation patterns within this network. Specifically, DBS reversed power spectra declines in SMN and increased abnormal functional connectivity within SMN. Power spectra describe the brain activity at 0.01–0.08 Hz in special brain networks, acting as an important biomarker reflecting intra-network intensities. These findings were consistent with previous studies. Horn et al. reported that DBS increased functional connectivity in the cerebello-thalamo-cortical network (Horn et al., 2019). Kahan et al. (2014) further clarified the DBS modulation effect on information flow within motor networks. STN-DBS inhibited the indirect pathway and excited the direct pathway, resulting in enhanced thalamic excitability, which increased primary motor cortex activity and accelerated the information flow in motor networks (Kahan et al., 2014, 2019).

For the inter-network differences, an interesting finding was that DBS was not effective to modulate the functional connectivity between different networks, but it was effective to modulate the functional connectivity between representative brain regions in each network. This suggested that DBS did not reverse the decoupling among networks in patients with PD but partially improved the inter-network connections by modulating the core brain regions within each network. This indicated the limitations of DBS for network modulation. Wu et al. noted that STN-DBS did not improve global network measures but was negatively associated with network assortativity (Wu et al., 2021), which consisted with our findings.

Another finding of the study was that the FPN also played an important role in DBS modulation. The functional connectivity between regions in FPN with other networks was modulated by DBS. The FPN is a top-down network, which allows the modulation of information processing from top to bottom in other brain regions to facilitate executive control and adaptive behavior (Dixon et al., 2018; Chen et al., 2021). In PD, the abnormal functional connectivity associated with FPN is usually inter-network (Tinaz, 2021). Similarly, we found that the abnormal functional connectivity in PMA/M1, r-IPL/r-PPC, l-dlPFC/mPFC, and PPC/ACC could be modulated by DBS. (1) DBS modulated the functional connectivity in PMA/M1, which reflected motor performance (Lam et al., 2018). Vervoort et al. reported that functional connectivity decreased between SMN and FPN in patients with PD when compared with controls (Vervoort et al., 2016). A dynamic functional network study also reported that the fractional window in FPN/SMN was reduced in patients with PD when compared with HCs, suggesting a reduced cross-talk between the two networks (Chen et al., 2021). This was closely associated with motor deficits in PD. Our results showed that DBS increased functional connectivity between the PMA and M1. This may increase the information transmission between networks, further improving motor symptoms. (2) DBS modulated the functional connectivity in the PPC/ACC, which reflected mood fluctuations (Lou et al., 2015). Hu et al. reported decreased functional connectivity between frontal-limbic regions in patients with PD with depression when compared with those without depression (Hu et al., 2015). Our results showed that DBS increased functional connectivity between PPC and ACC, which reversed the abnormal functional connectivity in patients with PD. (3) DBS modulated the functional connectivity in the regions of FPN/DMN, which was related to multiple neural events. Mohan et al. (2016) reported that abnormal changes between FPN and DMN were considered as disease-related network disruptions. Alterations in functional connectivity between FPN and DMN are associated with several non-motor symptoms in patients with PD, such as cognitive impairment (Lang et al., 2019), hallucination (Bejr-Kasem et al., 2019), and impulsive-compulsive behavior (Tessitore et al., 2017). Our results showed that DBS partially reversed functional connectivity between FPN and DMN. This might be the reason that DBS has efficacy in the treatment of PD non-motor symptoms. In summary, DBS demonstrated multi-network-modulated patterns with key nodes in SMN and FPN.

Our study identified the modulation of higher-order networks by DBS, which we speculated was mediated through the hyperdirect pathway (Polyakova et al., 2020). The hyperdirect pathway refers to direct projections between basal ganglia and cortex. The existence of a direct connection of fibers between these regions is the basis of this hypothesis. In PD, the hyperdirect pathway transmits excitatory stimuli from the motor, limbic, and associative brain regions (Quartarone et al., 2020). Electrophysiological studies confirm that β oscillation abnormalities are present in the basal ganglia-cortex circuits and are modulated by the STN-DBS (Oswal et al., 2021). Animal studies show that DBS can modulate motor control and mood disorders through hyperdirect pathway (Antonazzo et al., 2021; Nakata et al., 2021). These findings support the result of this study and provide a basis for further studies.

Previous research has shown that patients with PD had both ipsilateral and contralateral abnormal functional connectivities. Burman et al. (2014) reported that patients with PD showed a significant decrease in functional connectivity across the bilateral hemispheres of M1 when compared with HC. Furthermore, Fiorenzato et al. (2019) described brain networks in PD as “segregated,” recognizing that PD showed a significant weakening of intra- and inter-network connections when compared with HCs, which involved nearly all intrinsic networks. We found that almost all changes in multiple functional connectivities in patients with PD were reduced when compared with HCs, confirming the results of previous studies. To the best of our knowledge, the PD brain networks modulated by DBS were generally limited to the ipsilateral network, with less evidence of cross-hemispheric modulations. In this study, we noted that DBS only modulated ipsilateral abnormal functional connectivity. One possible explanation was that DBS induced an unbalanced modulation. Evidence has shown that bilateral DBS was asymmetric in the treatment of motor symptoms, leading to overstimulation on one side and understimulation on the other side (Kahan et al., 2014). Furthermore, Zhang et al. (2020) conducted asymmetric lead implantation, with STN on one side and globus pallidus internus (GPi) on the other side, producing satisfactory results. In summary, DBS demonstrated ipsilateral modulation patterns.

We further explored the correlations between the abnormal functional connectivity reversed by DBS and improvements in behavioral performance. It was interesting to note that the motor and depression were modulated by functional connectivity among different networks and that modulation heterogeneities existed even among different types of motor symptoms. (1) Improvement of all types of motor symptoms was related to functional connectivity changes within SMN and in FPN/SMN. The heterogeneity among symptoms may have arisen from neural remodeling caused by DBS, which gradually occurred. This process resulted in differences in improvement times among different motor symptoms (Krauss et al., 2004; de Hemptinne et al., 2015). Generally, tremor and rigidity improve in seconds to minutes, while axial often takes days to weeks to take effect (Ashkan et al., 2017). Moreover, Shen et al. (2020) reported that DBS modulated two distinct circles, namely, GPi and M1, which were associated with overall motor performance and bradykinesia, respectively. By employing volume tissue activation (VTA) as the seed, Akram et al. (2017) found that functional connectivity in VTA/SMA was correlated with rigidity and bradykinesia, while VTA/M1 was correlated with tremors. (2) Improvement of depression was related to functional connectivity changes in FPN/SN. SN, an important part of limbic circuits, is associated with mood control. A study on poststroke depression is consistent with our findings, which showed that the relationship of FPN/SN was closely related to the severity of depression (Shi et al., 2017). Furthermore, research of DBS-induced side effects shows that DBS changes functional connectivity from different brain regions, leading to different side effects, which also supports our idea that DBS modulation patterns have inter-symptom differences.

This study also had several limitations. First, patients included in this study might not picture the overall profile of PD, because DBS was mainly used for PD with the H&Y stage 2.5–4.0 (Bronstein et al., 2011). The patients with severe tremors were also excluded to cooperate with the MRI scan. Second, postoperative MRI is affected by magnetic field artifacts. To minimize the effect of artifacts on the results, we used ICA to separate RSNs. The selection of RSNs was based on sorting with templates obtained from published articles (Smith et al., 2009; Allen et al., 2011). All networks were identical to the previously reported distributions of healthy individuals. In addition, the selection of ROI strictly avoided the areas of artifacts. Moreover, the potentially affected areas, such as the left inferior parietal lobule (l-IPL), were excluded for the ROI analysis. Finally, we did not acquire preoperative fMRI data and failed to perform a longitudinal analysis. Future studies can consider this design, which will bring more benefits in understanding the changed processes of functional connectivity induced by DBS.

## Conclusion

This study found the modulation of DBS on multiple networks. Its changes were significantly correlated with the improvement of clinical symptoms. Specifically, DBS modulated the functional connectivity within SMN and across multiple networks centered by the FPN, and DBS was characterized by ipsilateral modulation patterns, with the patterns having inter-symptom differences. Overall, this study provided the basis for large-scale brain network research on multi-network DBS modulation.

## Data Availability Statement

The original contributions presented in the study are included in the article/Supplementary Material, further inquiries can be directed to the corresponding author/s.

## Ethics Statement

The studies involving human participants were reviewed and approved by IRB of Beijing Tiantan Hospital. The patients/participants provided their written informed consent to participate in this study.

## Author Contributions

YB, YDi, and YJ contributed to conceptualization, data curation, formal analysis, investigation, software, visualization, and writing – original draft. LG, ZZ, ZY, DC, HF, and QZ contributed to methodology, visualization, writing, reviewing, and editing. TH, HX, and DW contributed to data curation. YDu, FM, and YL contributed to supervision, project administration, writing, reviewing, and editing. JZ contributed to funding acquisition, project administration, resources, writing, reviewing, and editing. All authors contributed to the article and approved the submitted version.

## Conflict of Interest

The authors declare that the research was conducted in the absence of any commercial or financial relationships that could be construed as a potential conflict of interest.

## Publisher’s Note

All claims expressed in this article are solely those of the authors and do not necessarily represent those of their affiliated organizations, or those of the publisher, the editors and the reviewers. Any product that may be evaluated in this article, or claim that may be made by its manufacturer, is not guaranteed or endorsed by the publisher.

## Footnotes

1
https://www.fil.ion.ucl.ac.uk/spm

2
http://icatb.sourceforge.net/
