# Supplementary material for: Deep Brain Stimulation Modulates Multiple Abnormal Resting-State Network Connectivity in Patients With Parkinson’s Disease
Source: Front Aging Neurosci. 2022 Mar 21;14:794987. doi: 10.3389/fnagi.2022.794987 (PMC8978802; doi:10.3389/fnagi.2022.794987)
Supplement: Supplementary file 1 [file Data_Sheet_1.docx]

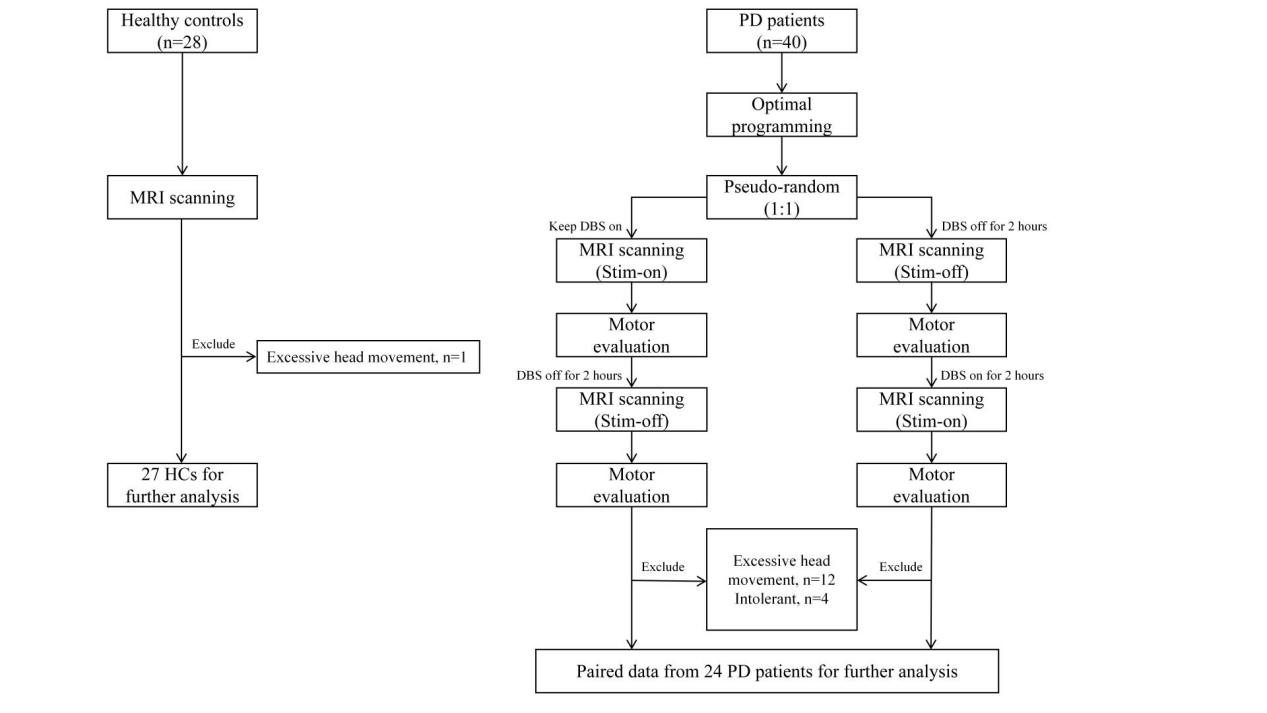


**Figure S1:**

The flow chart of this study.

**
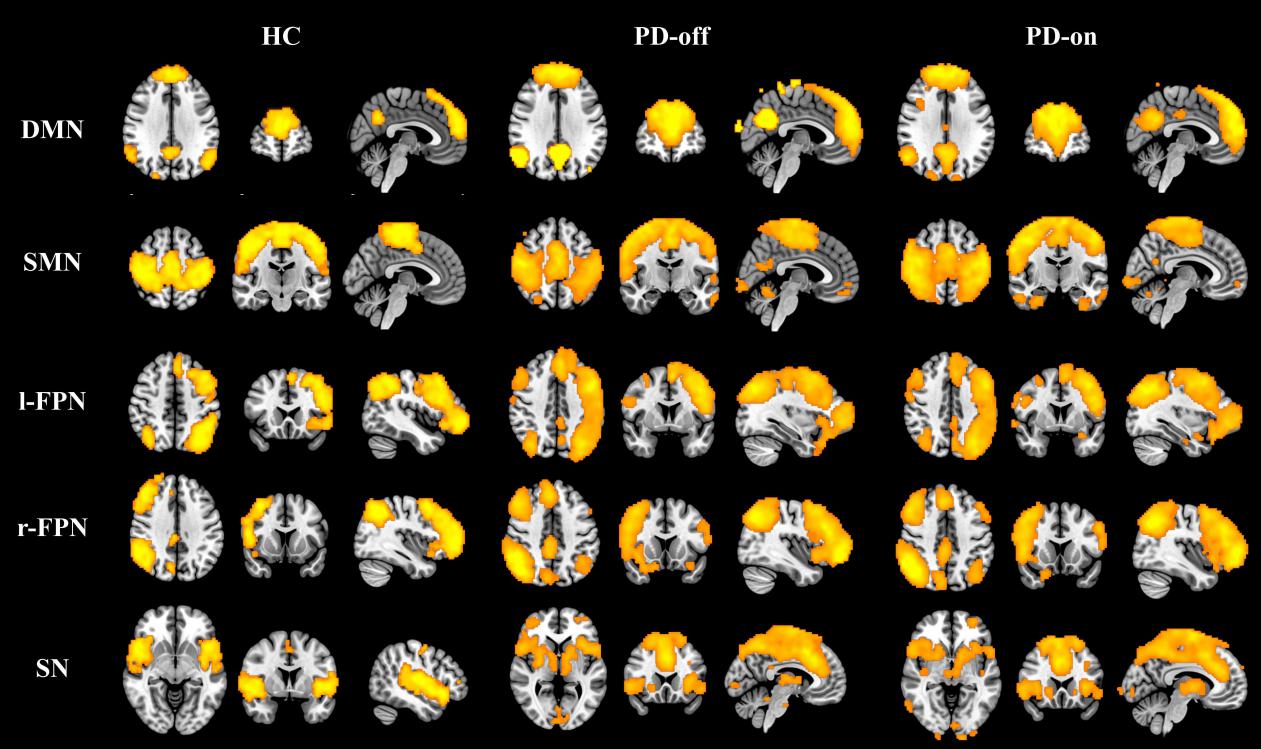
**

**Figure S2:**

Selected resting-state networks in different groups: group-level of HCs (left), group-level of PD with DBS off (PD-off, middle), group-level of PD with DBS on (PD-on, right). (FDR corrected, *P* < 0.05)

**
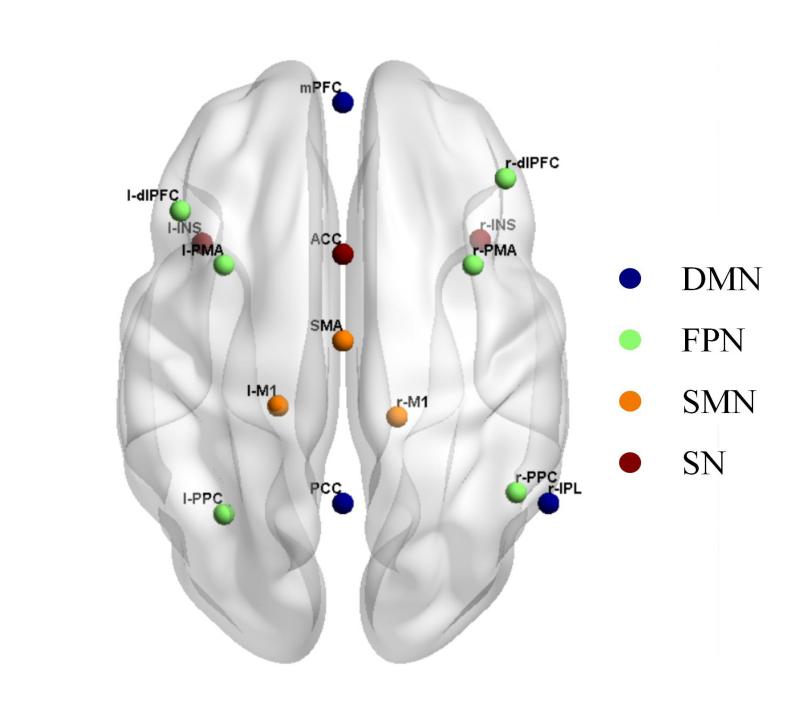
**

**Figure S3:**

Selected ROIs in the different resting-state networks (HC group as an example).

**
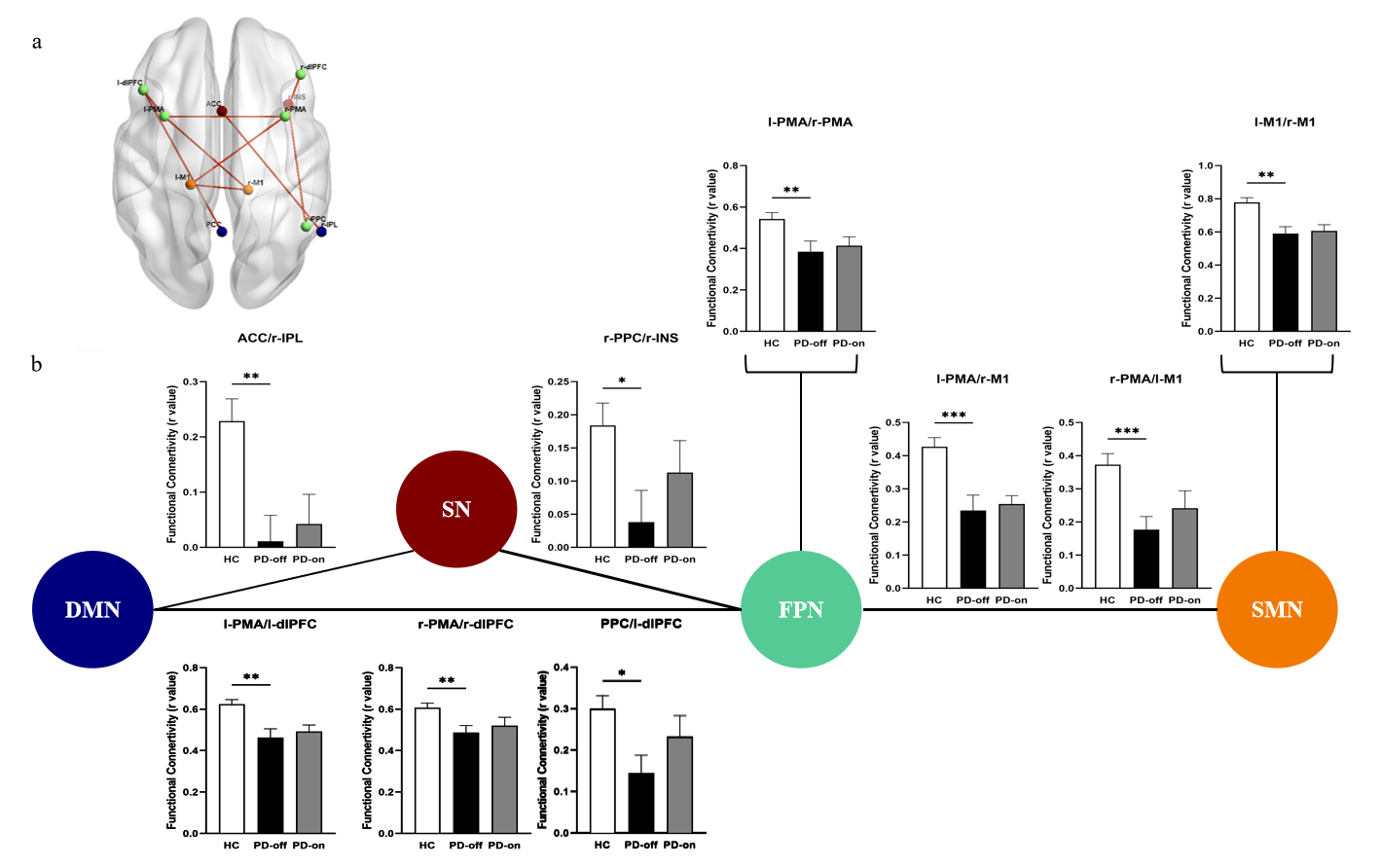
**

**Figure S4:**

1. Inter-states analyses of DBS unchanged abnormal functional connectivity in PD patients. Connections shown in light red represent HC > PD-off, no PD-off > HC result. (b) Additionally, these results were presented in bar graph among networks. Data represented as mean ± S.E. One-way ANOVA with post hoc test, FDR corrected, **P* < 0.05, ***P* < 0.01, ****P* < 0.001

**Table S1: Demographic and Clinical score in Individual PD patients**

| Patient No. | Age at surgery  (year) | Sex | Duration  (year) | Hoehn-Yahr stage | LED | Left electrode | | | | Right electrode | | | |
| --- | --- | --- | --- | --- | --- | --- | --- | --- | --- | --- | --- | --- | --- |
|  |  |  |  |  |  | Active Contact | Frequency (Hz) | Pulse Width (μs) | Voltage (volts) | Active Contact | Frequency (Hz) | Pulse Width (μs) | Voltage (volts) |
| 1 | 43 | Male | 8 | 3.0 | 1067 | 3- | 120 | 60 | 2.2 | 8- | 120 | 60 | 2.3 |
| 2 | 59 | Male | 17 | 3.0 | 1081 | 1- | 90 | 60 | 2.3 | 8- | 90 | 60 | 2.3 |
| 3 | 68 | Female | 13 | 3.0 | 851.2 | 2- | 130 | 60 | 1.8 | 10- | 130 | 60 | 1.8 |
| 4 | 73 | Female | 7 | 3.0 | 487.5 | 2- | 150 | 60 | 1.9 | 9- | 150 | 70 | 2.5 |
| 5 | 69 | Male | 16 | 4.0 | 2945 | 2- | 130 | 60 | 2.8 | 9- | 130 | 60 | 2.8 |
| 6 | 63 | Female | 6 | 2.5 | 500 | 2- | 155 | 80 | 2.7 | 10- | 155 | 70 | 2.4 |
| 7 | 60 | Male | 11 | 3.0 | 600 | 4- | 145 | 80 | 2.5 | 10- | 145 | 80 | 2.3 |
| 8 | 70 | Female | 10 | 3.0 | 675 | 1- | 145 | 90 | 3.0 | 9- | 145 | 80 | 1.8 |
| 9 | 59 | Male | 10 | 2.5 | 375 | 1- | 165 | 80 | 2.7 | 9- | 165 | 70 | 2.4 |
| 10 | 66 | Male | 12 | 2.5 | 644 | 3- | 130 | 60 | 1.5 | 11- | 130 | 60 | 1.5 |
| 11 | 60 | Male | 7 | 3.0 | 750 | 4- | 140 | 80 | 2.5 | 8- | 140 | 90 | 3.0 |
| 12 | 75 | Male | 10 | 2.5 | 250 | 2- | 145 | 80 | 2.4 | 10- | 145 | 80 | 2.2 |
| 13 | 69 | Female | 15 | 3.0 | 1000 | 3- | 145 | 100 | 2.5 | 10- | 145 | 90 | 2.8 |
| 14 | 64 | Male | 8 | 3.0 | 798 | 2- | 135 | 60 | 1.5 | 9- | 135 | 70 | 2.2 |
| 15 | 63 | Male | 11 | 3.0 | 900 | 1- | 135 | 60 | 3.1 | 9- | 135 | 60 | 2.6 |
| 16 | 62 | Female | 17 | 3.0 | 550 | 1- | 150 | 70 | 3.0 | 9- | 150 | 70 | 3.0 |
| 17 | 58 | Male | 9 | 3.0 | 873.8 | 2- | 145 | 70 | 2.3 | 11- | 145 | 70 | 2.3 |
| 18 | 47 | Male | 9 | 3.0 | 800 | 2- | 170 | 80 | 2.3 | 8- | 170 | 60 | 2.8 |
| 19 | 48 | Male | 9 | 3.0 | 200 | 2- | 145 | 70 | 2.0 | 9- | 145 | 60 | 2.6 |
| 20 | 65 | Male | 11 | 2.5 | 675 | 1- | 135 | 60 | 1.5 | 10- | 135 | 70 | 1.6 |
| 21 | 70 | Male | 15 | 2.5 | 750 | 3- | 155 | 70 | 2.2 | 8- | 155 | 70 | 2.3 |
| 22 | 64 | Female | 9 | 2.5 | 1351 | 1- | 90 | 60 | 1.4 | 9- | 90 | 60 | 1.5 |
| 23 | 59 | Female | 10 | 3.0 | 1000 | 1- | 160 | 80 | 2.5 | 9- | 160 | 70 | 2.0 |
| 24 | 66 | Female | 13 | 4.0 | 688 | 2- | 150 | 70 | 1.7 | 10- | 150 | 70 | 1.7 |

**Table S2: Comparison of DBS reversed functional connectivity among groups**

| Function Connectivity | HC vs PD-off | | | HC vs PD-on | | | PD-off vs PD-on | | |
| --- | --- | --- | --- | --- | --- | --- | --- | --- | --- |
|  | Difference | 95% CI | P value | Difference | 95% CI | P value | Difference | 95% CI | P value |
| SMA/l-M1^a^ | 0.18 | 0.10 - 0.34 | <0.001 | 0.12 | 0.05 - 0.22 | 0.006 | -0.11 | -0.22 - -0.01 | 0.020 |
| SMA/r-M1^a^ | 0.19 | 0.10 - 0.27 | <0.001 | 0.08 | 0.03 - 0.18 | 0.031 | -0.11 | -0.26 - -0.01 | 0.012 |
| l-PMA/l-M1 | 0.26 | 0.17 - 0.34 | <0.001 | 0.14 | 0.06 - 0.23 | 0.003 | -0.11 | -0.16 - -0.06 | 0.012 |
| r-PMA/r-M1 | 0.26 | 0.16 - 0.37 | <0.001 | 0.11 | 0.01 - 0.21 | 0.042 | -0.15 | -0.24 - -0.06 | 0.009 |
| mPFC/l-dlPFC | 0.19 | 0.07 - 0.30 | 0.001 | 0.07 | -0.01 - 0.15 | 0.125 | -0.11 | -0.22 - -0.01 | 0.031 |
| r-IPL/r-PPC | -0.14 | -0.24 - -0.04 | 0.014 | -0.04 | -0.14 - 0.07 | 0.349 | 0.11 | 0.03 - 0.18 | 0.021 |
| ACC/l-PPC | 0.14 | 0.02 - 0.26 | 0.011 | 0.02 | -0.09 - 0.14 | 0.673 | -0.11 | -0.21 - -0.01 | 0.027 |
| ACC/r-PPC | 0.15 | 0.03 - 0.26 | 0.009 | -0.03 | -0.14 - 0.08 | 0.223 | -0.18 | -0.29 - -0.06 | 0.006 |

P value were corrected by FDR

^a^ using nonparametric test
